# The Use of Gelatin Methacrylate (GelMA) in Cartilage Tissue Engineering: A Comprehensive Review

**DOI:** 10.3390/bioengineering12070700

**Published:** 2025-06-27

**Authors:** Kush Savsani, Alexandra Hunter Aitchison, Nicholas B. Allen, Elsie A. Adams, Samuel B. Adams

**Affiliations:** 1 School of Medicine, Virginia Commonwealth University, Richmond, VA 23298, USA; 2 Department of Orthopedic Surgery, Duke University Hospital, Durham, NC 27708, USA; 3 Department of Orthopedic Surgery, Durham VA Medical Center, Durham, NC 27705, USA

**Keywords:** GelMA, gelatin methacrylate, cartilage tissue engineering, 3D bioprinting, hybrid scaffolds

## Abstract

Cartilage injuries, due to their limited regenerative capacity, often result in chronic pain and functional impairment. These injuries are difficult to manage with conventional surgical repair techniques; therefore, alternative treatments are necessary. Gelatin methacrylate (GelMA) has emerged as a promising biomaterial for cartilage tissue engineering due to its biocompatibility, tunable mechanical properties, and ability to be used in advanced applications like 3D bioprinting. This review examines the synthesis, properties, and limitations of GelMA in cartilage repair, focusing on its applications in 3D bioprinting for the creation of patient-specific cartilage constructs. It also highlights preclinical studies exploring the potential of GelMA-based scaffolds in various animal models. Despite its advantages, challenges remain, such as the mechanical limitations of GelMA and its degradation rate in dynamic environments. Hybrid scaffolds, in situ bioprinting, and personalized bioinks offer solutions to these issues. Ultimately, long-term clinical trials are needed to assess the durability and efficacy of GelMA-based scaffolds in human applications. Future research is aimed at overcoming these challenges, improving the mechanical strength of GelMA scaffolds, and enhancing their clinical translation for cartilage repair.

## 1. Introduction

The limited self-healing potential of cartilage, due to its avascular nature and low cellularity, often leads to long-term pain, functional impairment, and joint degradation for patients. Traditional cartilage repair methods, including autologous chondrocyte implantation, microfracture surgery, and osteochondral autografts, often provide temporary relief but fail to fully restore the biomechanical properties of native cartilage [1,2,3]. These limitations have driven the need for more advanced therapeutic strategies such as those afforded by cartilage tissue engineering.

Tissue engineering aims to develop functional tissue substitutes by combining cells, scaffolds, and bioactive signals [4,5,6]. Biomaterials serve as scaffolds to support cell growth, differentiation, and tissue regeneration while mimicking the mechanical properties of native tissues. In recent years, a variety of natural and synthetic biomaterials have been explored for cartilage tissue engineering, including hyaluronic acid, chitosan, and polylactic-co-glycolic acid [7,8,9]. However, the ideal biomaterial for cartilage repair should provide a suitable environment for chondrocyte growth, exhibit tunable mechanical properties, and degrade over time in a manner that allows newly formed tissue to replace it.

Among these biomaterials, gelatin methacrylate (GelMA) has emerged as a promising candidate [10,11,12]. GelMA is derived from gelatin, a denatured form of collagen, and possesses favorable characteristics such as biocompatibility, tunability, and photocrosslinkability, making it ideal for use in tissue engineering. Importantly, GelMA’s versatility allows it to be applied in various forms, including hydrogels and bioinks, for 3D bioprinting. In particular, the ability to precisely control the architecture and composition of scaffolds through 3D bioprinting has opened new avenues for cartilage regeneration.

This review provides an in-depth examination of GelMA’s synthesis, properties, and applications in cartilage tissue engineering. We focus on its role in 3D bioprinting, summarize the findings from preclinical animal studies, and explore the potential for clinical translation. Additionally, we discuss the challenges and future perspectives for GelMA-based cartilage tissue engineering approaches.

## 2. Overview of GelMA Synthesis, Properties, and Limitations

### 2.1. Synthesis of GelMA

**Figure 1:**
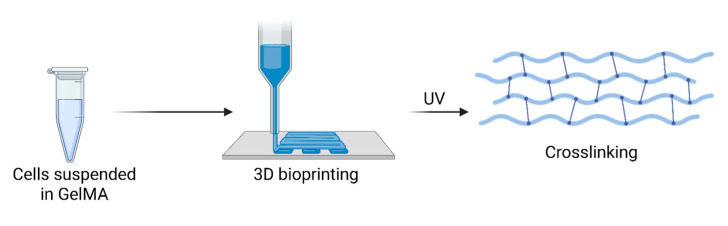
Biosynthesis of GelMA using 3D bioprinting.

GelMA is synthesized by modifying gelatin with methacrylic anhydride [13,14]. Gelatin, a natural biopolymer derived from collagen, contains bioactive sequences, such as arginine–glycine–aspartate (RGD) motifs, which promote cell adhesion and proliferation. Methacrylation introduces photoreactive methacryloyl groups into the gelatin structure, enabling the formation of covalent crosslinks when exposed to ultraviolet or visible light in the presence of photoinitiators. This photocrosslinking process results in the formation of hydrogels with tunable mechanical and biochemical properties. Figure 1 demonstrates the relationship between stages in GelMA synthesis.

The degree of methacrylation can be controlled during synthesis, allowing for precise customization of the resulting hydrogel’s mechanical stiffness, degradation rate, and cellular interactions [15,16,17]. Higher degrees of methacrylation result in stiffer hydrogels, whereas lower methacrylation degrees produce more flexible and degradable structures. By adjusting the methacrylation level, GelMA can be tailored to match the mechanical requirements of different tissues, including cartilage, which requires both stiffness and elasticity to withstand mechanical forces in the joint.

The versatility of GelMA extends beyond its mechanical properties, as it also enables the incorporation of bioactive molecules, growth factors, and other functional additives to enhance its biological performance. By fine-tuning the hydrogel formulation, researchers can create microenvironments that mimic native extracellular matrices, promoting specific cellular behaviors, such as proliferation, differentiation, and extracellular matrix deposition. Additionally, GelMA’s compatibility with various fabrication techniques, including 3D bioprinting and microfluidic systems, allows for the development of complex tissue constructs with spatially controlled architectures. These advantages make GelMA a highly adaptable biomaterial with broad applications in tissue engineering and regenerative medicine.

### 2.2. Key Properties of GelMA

GelMA hydrogels are highly advantageous for cartilage tissue engineering due to their exceptional biocompatibility and bioactivity. The presence of RGD motifs enhances chondrocyte attachment and facilitates extracellular matrix (ECM) deposition, which is necessary to form functional cartilage [18]. Additionally, the bioactive nature of GelMA enables it to promote cellular interactions and ECM production. Studies have demonstrated its ability to support the growth and differentiation of cell types, such as chondrocytes and mesenchymal stem cells (MSCs), making it an excellent candidate for engineering cartilage tissues [19].

GelMA demonstrates mechanical tunability by adjusting the degree of methacrylation and crosslinking in its synthesis [14]. This customization ensures the scaffold’s ability to withstand mechanical forces typical in load-bearing joints, an essential feature for clinical applications. Additionally, GelMA’s photocrosslinkable nature allows for precise control over scaffold architecture [20,21]. This capability facilitates the creation of complex, three-dimensional structures through advanced techniques like 3D bioprinting, enabling the fabrication of functional tissue constructs with spatial precision. GelMA scaffolds can be enzymatically degraded by cells, allowing the scaffold to be gradually replaced by newly formed tissue [22]. This property is integral to ensuring the long-term functionality of engineered cartilage.

GelMA hydrogels exhibit an ability to incorporate bioactive molecules, which can further enhance their functionality in cartilage regeneration. By integrating growth factors, cytokines, or other therapeutic agents within the hydrogel matrix, researchers can create a conducive environment that mimics the native cartilage niche. This modification not only supports the proliferation and differentiation of chondrocytes but also promotes angiogenesis and tissue integration. The versatility of GelMA in combining mechanical strength with biological cues makes it an ideal platform for advancing cartilage repair strategies, ultimately contributing to improved outcomes in tissue engineering applications.

### 2.3. Limitations of GelMA

While GelMA’s mechanical properties can be adjusted by varying its concentration or degree of methacrylation, it remains less robust compared to synthetic polymers, making it unsuitable for high-load-bearing applications such as load-bearing joints [23,24]. Incorporating reinforcing materials like nanomaterials or designing hybrid scaffolds that combine GelMA with other structural polymers may provide a pathway to enhancing its mechanical resilience [25].

Another limitation of GelMA is its reliance on photocrosslinking for hydrogel formation. Ensuring that photoinitiators are non-toxic and biocompatible over the long term is crucial, as any residual photoinitiator toxicity could hinder tissue regeneration or lead to adverse cellular responses [26]. Additionally, the necessity for precise light delivery in vivo may complicate procedures, especially in deep or irregularly shaped tissues. Strategies to optimize photoinitiators or explore alternative crosslinking mechanisms could help mitigate these challenges and expand the applicability of GelMA in clinical environments.

The limited long-term stability of GelMA in biomedical applications may be another limitation. While its biodegradability is an advantage for transient scaffolds, it can degrade too quickly in dynamic environments like joints, where mechanical and enzymatic stresses are high. This rapid degradation risks compromising the structural integrity and functionality of engineered cartilage over time. To address this, research efforts are focusing on tuning GelMA’s degradation rate by modifying its crosslink density or incorporating protective additives [27]. Enhancing its resistance to enzymatic breakdown without sacrificing biocompatibility will be essential for achieving sustained efficacy in cartilage repair.

## 3. GelMA in 3D Bioprinting for Cartilage Tissue Engineering

### 3.1. Advantages of 3D Bioprinting with GelMA

Three-dimensional bioprinting is a cutting-edge technology that enables the precise deposition of bioinks to create complex tissue constructs with spatial control over scaffold architecture. This approach is particularly well-suited for cartilage tissue engineering, where the ability to replicate the intricate structure and mechanical properties of cartilage is critical for successful tissue regeneration. GelMA’s photocrosslinking capability makes it an ideal candidate for 3D bioprinting, as it allows for the fabrication of highly detailed scaffolds with tunable mechanical properties.

One of the key advantages of using GelMA in 3D bioprinting is its versatility. GelMA-based bioinks can be customized to match the mechanical properties of native cartilage, promoting the development of functional cartilage tissue [27]. Additionally, GelMA supports the encapsulation of a wide range of cell types, including chondrocytes, stem cells, and other progenitor cells [19]. This versatility makes GelMA an attractive bioink for fabricating patient-specific cartilage constructs.

GelMA’s biocompatibility and biodegradability enhance its appeal for cartilage tissue engineering. As a naturally derived polymer, GelMA facilitates cellular adhesion, proliferation, and differentiation, which are essential for successful tissue integration and functionality. Its ability to degrade over time aligns with the natural remodeling processes of tissue, allowing for the gradual replacement of the scaffold with newly formed tissue. This dynamic interaction between the scaffold and the cells can lead to improved tissue quality and functionality, ultimately contributing to more effective regenerative therapies for cartilage repair.

### 3.2. GelMA-Based Bioinks for Cartilage Repair

GelMA has shown efficacy as a bioink for cartilage bioprinting due to its excellent biocompatibility, tunable mechanical properties, and ability to support cell growth and differentiation. Studies have demonstrated that GelMA-based bioinks can effectively promote chondrogenesis and support the formation of cartilage-like tissue both in vitro and in vivo. Yin et al. investigated the physical properties and cellular responses of GelMA hydrogels, finding high compatibility with cellular behavior and its ability to create suitable environments for cartilage regeneration [28]. Zhang et al. explored a TGF-β-loaded GelMA hydrogel scaffold designed to promote chondrogenic differentiation, demonstrating the chondrogenic potential of GelMA-based bioinks [29].

The inclusion of bioactive factors such as growth factors or signaling molecules further enhances the chondrogenic potential of GelMA-based bioinks. Researchers have incorporated factors such as TGF-β3 and BMP-2 into GelMA bioinks to drive chondrogenesis and improve cartilage tissue formation [29,30]. Such approaches enable the creation of bioengineered constructs with enhanced ECM production and mechanical properties, making GelMA a promising material for advancing cartilage regeneration therapies.

The versatility of GelMA bioinks allows for the incorporation of various cell types, including mesenchymal stem cells and chondrocytes, which can significantly influence regenerative outcomes. The combination of GelMA with these cell types can improve cell viability and promote a more organized tissue structure. This also enhances the mechanical properties of the engineered cartilage and facilitates nutrient and waste exchange within the tissue construct. The adaptability of GelMA formulations, which can be modified for specific applications, makes it an attractive choice for creating personalized cartilage repair solutions tailored to individual patient needs.

Advancements in bioprinting techniques have enabled precise spatial control over cell distribution and biomaterial composition within GelMA constructs. This level of control allows researchers to mimic the native cartilage microenvironment more closely, promoting optimal cell behavior and tissue development. As technologies such as multi-material bioprinting and dynamic mechanical loading continue to evolve, the potential for GelMA-based bioinks in cartilage tissue engineering is likely to expand, paving the way for innovative therapies that could improve patient outcomes in cartilage repair and regeneration.

### 3.3. Hybrid Bioinks for Enhanced Cartilage Regeneration

One of the limitations of GelMA for cartilage bioprinting is its relatively low mechanical strength, which may not be sufficient for load-bearing applications. To address this challenge, researchers have developed hybrid bioinks by combining GelMA with other materials to enhance the mechanical and bioactive properties of the printed scaffolds. Zheng et al. developed sequentially cross-linked bioactive glass/GelMA composite hydrogels for tissue engineering applications, which exhibited enhanced mechanical properties and bioactivity, demonstrating their potential for promoting tissue regeneration [31].

Mendes et al. investigated the addition of graphene oxide to photocrosslinkable GelMA hydrogels, which showed significant improvements in electroactivity, mechanical strength, and printability [32]. Similarly, Shin et al. designed hybrid hydrogels that incorporated graphene oxide into GelMA [33]. These hybrid gels demonstrated enhanced mechanical properties in addition to supporting chondrocyte viability and ECM production, making them promising for cartilage repair. Table 1 outlines key differences in the physical properties of native GelMA with common hybrid scaffolds. The incorporation of bioactive nanoparticles or other reinforcing materials makes hybrid bioinks more suitable for load-bearing applications while simultaneously enhancing their bioactivity to promote efficient cartilage regeneration.

### 3.4. Printability and Crosslinking Strategies for GelMA-Based Constructs

The printability of GelMA bioinks is a crucial factor influencing the success of 3D bioprinting for cartilage tissue engineering. Various factors, including viscosity, crosslinking mechanisms, and extrusion parameters, impact the structural integrity and mechanical properties of the printed constructs [35,36,37]. The degree of methylacrylation in GelMA affects its crosslinking efficiency and mechanical properties, which allows for the precise modification of unique applications. Additionally, dual-crosslinking strategies have been utilized to enhance the mechanical strength and durability of GelMA scaffolds [38,39,40].

Studies have found that the balance between viscosity and shear-thinning behavior plays a crucial role in maintaining printability while ensuring cell viability during extrusion-based bioprinting [35]. The inclusion of bioactive fillers, such as hydroxyapatite or tannic acid, has been explored to improve the stability and functionality of GelMA-based bioinks while preserving their biocompatibility [36]. Additionally, hybrid formulations that incorporate collagen or sulfhydrylated chitosan have demonstrated enhanced mechanical properties and bioactivity, making them suitable for complex tissue engineering applications, including osteochondral repair [37,38,39]. The integration of advanced crosslinking mechanisms and hybrid bioink formulations continues to drive the development of next-generation GelMA scaffolds tailored for cartilage tissue engineering.

### 3.5. Cell-Laden GelMA Constructs for In Vivo Regeneration

Successful cartilage regeneration requires not only biomimetic scaffold structures but also the incorporation of viable cells capable of producing extracellular matrix components. Cell-laden GelMA constructs have been extensively studied for their ability to support chondrocyte and stem cell proliferation, differentiation, and ECM deposition. Studies have demonstrated that pre-differentiating stem cells within GelMA hydrogels before implantation can enhance in vivo chondrogenesis and improve integration with native cartilage tissue [41,42,43]. Additionally, co-culture strategies utilizing multiple cell types, such as chondrocytes and mesenchymal stem cells, have been explored to enhance matrix production and accelerate cartilage repair [23,44,45].

The long-term viability and functional performance of encapsulated cells within GelMA hydrogels are influenced by factors such as hydrogel stiffness, porosity, and degradation rate. Studies have shown that tuning these properties can significantly impact cell survival, proliferation, and differentiation [41]. Modifying the degree of methacrylation in GelMA can regulate the stiffness of the hydrogel, thereby influencing stem cell fate and promoting lineage-specific differentiation [42]. Additionally, the incorporation of bioactive components, such as growth factors or hybrid biomaterials, has been explored to enhance cell–matrix interactions and improve tissue maturation [43]. Microfluidic-assisted bioprinting and dynamic culture systems have further been utilized to optimize nutrient diffusion and mechanical stimulation within GelMA scaffolds, leading to improved chondrogenic outcomes [44,45].

## 4. Practical Considerations

### 4.1. Scalability and Manufacturing Challenges

Scalability and manufacturing present significant challenges for producing GelMA-based scaffolds at a clinical scale. Consistency, reproducibility, and quality control are critical issues that must be addressed to ensure that GelMA scaffolds meet the rigorous standards required for clinical applications. Optimizing manufacturing processes—such as automating synthesis, standardizing photo crosslinking protocols, and implementing real-time quality monitoring—is essential for the reliable production of GelMA-based constructs.

To further enhance the scalability of GelMA scaffold production, collaboration between researchers, industry partners, and regulatory bodies will be crucial. Engaging in multidisciplinary approaches that incorporate engineering principles, materials science, and clinical insights can lead to innovative solutions that streamline production workflows and enhance scaffold performance. Additionally, exploring novel manufacturing techniques, such as large-scale 3D bioprinting and microfabrication, may offer new avenues for creating complex, patient-specific constructs that align with the evolving demands of regenerative medicine.

### 4.2. Sustainability and Regulatory Considerations

For GelMA-based therapies to successfully transition from bench to bedside, considerations of sustainability and regulatory compliance are equally critical. Scalable production must not only be economically viable but also environmentally sustainable. This includes the utilization of renewable resources and the development of cost-effective manufacturing processes.

One concern is the reliance on animal-derived gelatin, typically porcine or bovine collagen, which raises issues related to resource renewability, ethical sourcing, and batch-to-batch variability. Additionally, the use of photoinitiators and crosslinking agents, which may be toxic or non-biodegradable, such as Irgacure 2959 and lithium phenyl-2-4-6-trimethylbenzophosphinate, can contribute to environmental and health risks if not properly managed. Solvent usage and energy-intensive synthesis processes further add to the sustainability challenge.

Standardization of synthesis protocols and fabrication methods is vital for ensuring reproducibility and meeting the stringent quality-control requirements imposed by regulatory agencies. A lifecycle assessment of GelMA-based therapies should be integrated into the development process to evaluate their environmental impact from production to disposal. Emphasizing biocompatibility and biodegradability in the design of GelMA materials can enhance their appeal, as these properties align with the growing demand for eco-friendly medical solutions.

### 4.3. Storage and Shelf-Life Stability

The stability of GelMA-based bioinks and scaffolds during storage is a crucial factor in their practical application. Variables such as temperature, humidity, and light exposure can affect the integrity and performance of GelMA hydrogels, potentially impacting their mechanical properties and bioactivity [46,47]. Freeze-drying and lyophilization have been explored as methods to enhance the shelf-life of GelMA, allowing for long-term storage without significant degradation [48,49]. However, rehydration and sterilization protocols must be carefully optimized to maintain the bioink’s printability and biocompatibility. The development of stable, ready-to-use GelMA formulations that can be stored at ambient or refrigerated conditions would greatly improve their practicality for clinical and industrial applications.

### 4.4. Cost and Economic Feasibility

The high production cost of GelMA-based scaffolds remains a significant barrier to large-scale adoption in tissue engineering and regenerative medicine. Factors such as the cost of raw materials, synthesis complexity, and bioprinting infrastructure contribute to the overall expense of GelMA-based constructs. To make these technologies more accessible, cost-effective synthesis strategies, such as enzymatic crosslinking alternatives and bulk-scale production techniques, need to be explored [50,51]. Additionally, the integration of automation in biofabrication processes can reduce labor costs and improve throughput. Economic feasibility studies comparing GelMA-based approaches to existing cartilage repair treatments are essential in determining their potential for widespread clinical implementation.

Organizations should look to leverage the potential long-term cost savings associated with GelMA-based scaffolds. These advanced materials not only aim to improve patient outcomes by promoting more effective tissue regeneration but also have the capacity to reduce the frequency of revision surgeries and associated healthcare expenses. As GelMA technology matures and clinical data on its effectiveness becomes more robust, healthcare providers may find that investing in these innovative solutions could lead to lower overall costs in patient management. The focus on demonstrating value through improved clinical outcomes will be crucial in persuading stakeholders to embrace GelMA-based scaffolds as a viable alternative to traditional treatment options.

## 5. Discussion

GelMA has emerged as a leading biomaterial in cartilage tissue engineering due to its biocompatibility, tunable mechanical properties, and ability to support chondrogenesis. Its retention of collagen-derived bioactive motifs enhances cell adhesion and extracellular matrix deposition, facilitating neocartilage formation. The photocrosslinkable nature of GelMA allows for precise control over scaffold architecture, making it highly compatible with 3D bioprinting and enabling the fabrication of patient-specific constructs. These properties position GelMA as a strong candidate for cartilage repair, but significant challenges remain.

Mechanical instability is a primary limitation, particularly in load-bearing applications. Even with optimized methacrylation and crosslinking density, GelMA hydrogels lack the stiffness and toughness of native cartilage. Compression testing in preclinical models consistently shows that GelMA alone does not achieve the mechanical resilience required for high-stress environments. Attempts to improve structural integrity through increased polymer concentration often result in reduced porosity and hindered nutrient diffusion, which can negatively impact chondrocyte viability and matrix deposition. These trade-offs must be addressed before GelMA can be widely adopted for high-load applications.

The degradation rate is another critical challenge. While controlled degradation is necessary to allow for tissue remodeling, GelMA hydrogels degrade too rapidly under enzymatic and mechanical stress in joint environments, leading to premature loss of scaffold integrity. In vivo studies have reported accelerated hydrogel breakdown, limiting the time frame for effective cartilage regeneration. Modifications, such as increased crosslinking density or the incorporation of enzyme-resistant polymers, have shown some promise in extending scaffold longevity, but maintaining a balance between degradation and tissue formation remains a major design constraint.

Clinical translation is still in its early stages, with most studies limited to in vitro and small-animal models. While these models demonstrate GelMA’s potential for chondrocyte proliferation and matrix production, large-animal studies are necessary to evaluate long-term integration and mechanical function under physiological loading. Additionally, standardizing GelMA synthesis and fabrication processes is critical for ensuring reproducibility and regulatory approval. Differences in methacrylation degree, crosslinking conditions, and photoinitiator selection can significantly alter mechanical properties and degradation profiles, highlighting the need for stringent quality control before clinical application.

Addressing these issues will determine whether GelMA advances from an experimental platform to a viable clinical solution for cartilage repair, and interdisciplinary collaboration among materials scientists, bioengineers, and clinicians will be crucial in overcoming these challenges. Such collaboration can facilitate the optimization of GelMA formulations, the integration of advanced manufacturing techniques, and the development of scalable production processes that meet regulatory standards.

## 6. Future Directions

The field of GelMA-based cartilage tissue engineering is rapidly evolving with several areas of research that could significantly enhance the clinical utility of GelMA scaffolds. Future work should aim to improve mechanical performance, develop personalized therapies, and create systems that actively respond to local tissue conditions.

### 6.1. Hybrid and Reinforced Scaffolds

The development of hybrid scaffolds that combine GelMA with other materials, such as synthetic polymers or bioactive nanoparticles, is critical for enhancing the mechanical strength of GelMA-based constructs, particularly for load-bearing applications like knee cartilage repair. By incorporating stiffer materials or bioactive components, hybrid scaffolds could provide the necessary structural support while maintaining the biocompatibility and bioactivity of GelMA. Among the promising materials for hybrid scaffolds are graphene and graphene oxide, which have shown potential in improving the mechanical properties and electrical conductivity of GelMA scaffolds [32,33]. These graphene-reinforced scaffolds could not only enhance the mechanical strength required for cartilage repair but may also promote cartilage regeneration by stimulating chondrocyte activity and ECM deposition. Additionally, bioactive nanoparticles, such as bioactive glass or hydroxyapatite, could further enhance the mechanical and biological properties of GelMA-based scaffolds [31], making them more suitable for complex cartilage and osteochondral repair.

The integration of these advanced materials into hybrid scaffolds opens new avenues for tailored tissue engineering applications. For instance, the combination of GelMA with polycaprolactone (PCL) offers a favorable balance of elasticity and strength, enabling the development of scaffolds that can withstand dynamic mechanical loads typical of joint movement. Furthermore, incorporating PCL can improve the degradation rate of the scaffold, allowing for a more synchronized tissue regeneration process. This synergy between materials not only optimizes mechanical properties but also enhances cell attachment and proliferation, which are crucial for effective cartilage repair.

Additionally, the versatility of hybrid scaffolds allows for the incorporation of growth factors or other therapeutic agents that can facilitate tissue healing and regeneration. By embedding these bioactive substances within the scaffold matrix, researchers can create a localized environment that supports cellular activities essential for cartilage formation. The controlled release of these factors over time can further enhance the scaffold’s regenerative capabilities, potentially leading to improved outcomes in cartilage repair strategies.

### 6.2. In Situ Bioprinting and Personalized Bioinks

The advancement of in situ bioprinting technologies offers the potential for the direct repair of cartilage defects during surgery. In situ bioprinting involves the deposition of bioinks directly onto tissue defects, improving scaffold placement and promoting immediate tissue repair. Laser-assisted bioprinting is one such example that has shown promise in achieving high print fidelity and architectural precision during bioink deposition. This technology could be adapted for in situ cartilage bioprinting, facilitating the creation of patient-specific cartilage constructs during surgery.

The development of personalized bioinks offers another avenue of patient-specific treatment options for GelMA-based cartilage repair. The incorporation of autologous chondrocytes, stem cells, or extracellular matrix components into GelMA scaffolds could improve scaffold integration and reduce the risk of immune rejection. This tailored approach would enhance the success of cartilage regeneration therapies by addressing the specific needs of individual patients.

### 6.3. Smart Scaffolds and Multifunctional Systems

Beyond serving as a passive structural support, GelMA has the potential to be engineered into “smart” scaffolds that respond dynamically to the tissue environment. By incorporating stimuli-responsive elements—such as thermoresponsive, pH-sensitive, or enzymatically degradable groups—these scaffolds could alter their behavior in real time. Analogous strategies are already being employed in related hydrogel systems. Such modifications to GelMA scaffolds may allow for controlled, localized drug delivery or the on-demand release of growth factors, thereby actively supporting the healing process by reducing inflammation and promoting tissue regeneration.

### 6.4. Integration with Emerging Technologies

New technologies offer additional opportunities to enhance GelMA-based scaffolds. Linking these scaffolds with advanced imaging techniques and biosensors could allow for the real-time monitoring of scaffold integration and tissue repair. In parallel, computational modeling and machine learning may help optimize scaffold design and predict in vivo performance, which will contribute to the development of more personalized and effective regenerative treatments.

The combination of GelMA scaffolds with 3D bioprinting techniques has the potential to revolutionize tissue engineering. By precisely controlling the spatial arrangement of cells and biomaterials, researchers can create scaffolds that mimic the natural extracellular matrix more closely. This level of customization can improve cell proliferation, differentiation, and overall tissue functionality. Additionally, the ability to incorporate multiple cell types and bioactive molecules within the printed structures can enhance the regenerative capacity of the scaffolds, leading to better outcomes in tissue repair and regeneration.

Additionally, integrating GelMA scaffolds with smart materials that respond to environmental stimuli could further enhance their functionality. For example, incorporating shape-memory polymers or hydrogels that change properties in response to pH, temperature, or light could facilitate controlled drug release or enhance cellular responses. These innovations would enable more dynamic interactions between the scaffold and the surrounding tissue, potentially leading to more effective healing processes. As these technologies continue to evolve, the potential applications for GelMA-based scaffolds in regenerative medicine will expand, paving the way for novel therapeutic strategies.

### 6.5. Long-Term Clinical Evaluation

Long-term clinical studies are essential to assess the durability, mechanical stability, effectiveness, and functional outcomes of GelMA-based scaffolds in patients. A study involving 3D-printed GelMA scaffolds reinforced with silk fibroin and hydroxyapatite reported a 2.5-fold increase in comprehensive modulus compared to pure GelMA, with values rising from approximately 50 kPa to 125 kPa. These hybrid scaffolds also supported enhanced chondrocyte proliferation and differentiation, with sustained cell viability and scaffold integrity observed over a 28-day in vitro period. Such findings suggest strong potential for long-term in vivo applications [52].

To facilitate clinical translation, pilot human trials should incorporate patient-reported outcome measures, such as the Knee injury and Osteoarthritis Outcome Score (KOOS), arthroscopic evaluations to directly observe scaffold performance, and biomarker tracking (e.g., cartilage oligomeric matrix protein or CTX-II) to monitor cartilage turnover. A robust post-implantation monitoring framework is also essential. This should include scheduled clinical and imaging assessments at intervals such as 3, 6, 12, and 24 months post-surgery, along with adverse-event tracking to identify complications, like scaffold disintegration, immune rejection, or joint instability. In cases requiring revision surgery, biopsy or explant analysis can provide valuable insights into failure mechanisms.

Furthermore, the development of a robust follow-up strategy is crucial for capturing any late-onset complications or failures associated with GelMA-based therapies. A well-defined framework for post-implantation monitoring can aid in identifying issues such as scaffold degradation, mechanical failure, or adverse tissue reactions that may arise over time. A proactive approach will not only enhance patient safety but also refine the design and application of GelMA scaffolds in clinical settings, ultimately paving the way for improved treatment modalities in regenerative medicine.

## 7. Conclusions

GelMA has shown great potential in the field of cartilage tissue engineering, offering an adaptable and biocompatible platform for creating scaffolds that support cartilage regeneration. Its mechanical properties, which can be tuned through synthesis, and its ability to facilitate cell growth and ECM deposition make it a promising candidate for cartilage repair. However, challenges such as its relatively low mechanical stability and rapid degradation in dynamic environments remain significant barriers to its clinical application, particularly for high-load-bearing joints like the knee. Ongoing efforts to develop hybrid scaffolds, incorporate bioactive nanoparticles, and improve in situ bioprinting techniques are crucial for overcoming these limitations. Personalized bioinks, tailored to individual patients, offer a promising approach to improving scaffold integration and reducing immune rejection. While preclinical animal studies have demonstrated the efficacy of GelMA-based scaffolds in promoting cartilage regeneration, long-term clinical studies are essential to assess the durability, functional outcomes, and overall safety of these scaffolds in human patients. Continued advancements in GelMA-based technology, alongside optimized manufacturing and regulatory processes, will be key to unlocking the full clinical potential of GelMA in cartilage repair.

## Figures and Tables

**Table 1:** Comparison of native GelMA properties with common hybrid scaffolds [34].

Property	GelMA	Hybrid Scaffolds
Stiffness	Tunable by methacrylation degree. Typically ranges from 1 to 50 kPa.	Enhanced with reinforcing materials like graphene oxide. GelMA + Alginate + Gelatin has shown mechanical strength of 100 kPa.
Tensile Strength	Limited. Not suitable for high-load applications. Initial tensile strength is around 0.4 kPa.	Improved with hybrid materials. GelMA + Graphene Oxide showed tensile strength of 1100 kPa after mechanical training and crosslinking.
Degradation Rate	Rapid in dynamic environments. Degeneration usually takes about 1–2 weeks.	Controlled with crosslinking density and protective additives. The inclusion of bioactive glass allows for controlled degradation, taking 4–6 weeks.

## Abbreviations

The following abbreviations are used in this manuscript:

GelMA	Gelatin methacrylate
RGD	Arginine–glycine–aspartate
ECM	Extracellular matrix
MSCs	Mesenchymal stem cells
PCL	Polycaprolactone
TGF-B	Transforming growth factor-beta
BMP-2	Bone morphogenetic protein 2
